# The Chikungunya Virus nsP3 Macro Domain Inhibits Activation of the NF-κB Pathway

**DOI:** 10.3390/v17020191

**Published:** 2025-01-29

**Authors:** Grace C. Roberts, Nicola J. Stonehouse, Mark Harris

**Affiliations:** School of Molecular and Cellular Biology, Faculty of Biological Sciences and Astbury Centre for Structural Molecular Biology, University of Leeds, Leeds LS2 9JT, UK; g.c.roberts@leeds.ac.uk (G.C.R.); n.j.stonehouse@leeds.ac.uk (N.J.S.)

**Keywords:** chikungunya virus, nsP3, macro domain, ADP-ribose, NF-κB

## Abstract

The role of the chikungunya virus (CHIKV) non-structural protein 3 (nsP3) in the virus lifecycle is poorly understood. The protein comprises three domains. At the N-terminus is a macro domain, biochemically characterised to bind both RNA and ADP-ribose and to possess ADP-ribosyl hydrolase activity—an enzymatic activity that removes ADP-ribose from mono-ADP-ribosylated proteins. As ADP-ribosylation is important in the signalling pathway, leading to activation of the transcription factor NF-κB, we sought to determine whether the macro domain might perturb NF-κB signalling. We first showed that CHIKV infection did not induce NF-κB activation and could not block exogenous activation of the pathway via TNFα, although TNFα treatment did result in a modest reduction in virus titre. In contrast, ectopic expression of nsP3 was able to inhibit both basal and TNFα-mediated NF-κB activation, and this was dependent on the macro domain, as a mutation previously shown to disrupt ADP-ribose binding and hydrolase activity (D10A) eliminated the ability to inhibit NF-κB activation. The macro domain D10A mutant also resulted in a dramatic reduction in virus infectivity, consistent with the notion that the ability of the macro domain to inhibit NF-κB activation plays a role in the virus lifecycle.

## 1. Introduction

Chikungunya virus (CHIKV) is an alphavirus of the *Togaviridae* family [1] and is increasingly becoming a threat to global health. First discovered in Tanzania in 1952, CHIKV is now classified into four distinct genotypes and has spread from Africa to Asia, Europe, and the Americas [2]. CHIKV is transmitted by mosquitos and was previously geographically limited to the distribution of the *Aedes aegypti* mosquito, which favours tropical climates. However, mutations in the E1 and E2 glycoproteins, detected in 2004, have enabled spread by the *Aedes albopictus* mosquito, which is more widely disseminated around the globe [3].

Infection with CHIKV causes Chikungunya fever, characterised by high fever, rash, and joint pain which can be debilitating and, in up to 50% of cases, can persist for many years after infection [4]. CHIKV infection induces a highly inflammatory state, inducing tissue damage [5]. In addition, individuals who have been infected with CHIKV are significantly predisposed to arthritis later in life [6]. In healthy adults, CHIKV is rarely fatal, although the risk of death is high in infants, the elderly, and the immunocompromised [7].

The CHIKV genome is a positive-sense, single-stranded (ss) RNA, with a 5′ cap, a poly-A tail, and containing two open reading frames (ORFs) [8]. The first ORF encodes for the four non-structural proteins, nsP1-4, which are directly translated from the genomic RNA [9]. The second ORF encodes for the structural proteins and is translated from a sub-genomic RNA, which is generated from a negative sense template following genome replication [10]. The functions of nsP1, 2, and 4 have been well defined: nsP1 displays both capping and decapping activities [11] and forms a dodecameric ring at the entrance to the membrane-bound spherules where RNA replication takes place [12]. NTPase, helicase, and protease activities are provided by nsP2, the latter being responsible for the processing of the non-structural polyprotein [13,14]. NsP4 is the RNA-dependent RNA polymerase (RdRp) [15].

Despite numerous studies, the role of nsP3 remains to be unambiguously defined. In addition to a role during genome RNA replication and the interactions with a range of viral and cellular proteins, it is likely to play multiple roles in the CHIKV lifecycle [16]. NsP3 comprises three domains: at the N-terminus is the macro domain, followed by the alphavirus unique domain (AUD), and the hyper-variable domain (HVD) (Figure S1a). The macro domain is found in the proteins of bacteria, eucaryotes, archaea, and positive-sense RNA viruses [17]. This domain has been shown to interact with, and hydrolyse, ADP-ribose from ADP-ribosylated proteins. Although it has been shown that macro domains of different species can differ greatly in their affinity for ADP-ribose and enzymatic activity, the CHIKV macro domain has been demonstrated to both bind ADP-ribose [18] (Figure S1b) and possess hydrolase activity [19]. It has been shown that these two properties of the CHIKV macro domain are essential for virus replication in cell culture [20]. Recently, it was also demonstrated that the nsP3 macro domain reverses the mono-ADP-ribosylation (MARylation) of nsP2 by ARTD10 (a mono-ADP-ribosylating enzyme, also known as PARP10) [21]. MARylation of nsP2 by this IFN-stimulated enzyme has been shown to be anti-viral, as it restricts nsP2 proteolytic activity, resulting in impaired viral polyprotein processing. The AUD is a less well understood domain, found to be essential for RNA replication but has also been implicated in virus assembly [22]. The HVD varies greatly in both sequence and size between the alphaviruses and has been more widely studied [23]. This intrinsically disordered and unstructured domain is highly phosphorylated [24] and facilitates the majority of known protein interactions with nsP3 [16]. Substitution of phosphorylated serine and threonine residues in the CHIKV nsP3 HVD resulted in reduced replication and infectivity.

Although CHIKV has been shown to induce a robust interferon (IFN) response in infected cells, which is crucial for an appropriate immune response and for survival in infected mice [25], there has been little characterisation of the other innate immunity pathways in response to CHIKV infection. In particular, the interplay between CHIKV infection and the NF-κB pathway remains largely uncharacterised. One study proposed that CHIKV infection suppressed activation of NF-κB by increasing the expression of the micro-RNA miR-146a, which in turn downregulated the expression of the upstream modulators TRAF6, IRAK1, and IRAK2 [26]. Another study demonstrated that various CHIKV proteins, particularly nsP2, have inhibitory effects on MDA5 and RIG-I, both of which are dsRNA sensors that can activate the NF-κB pathway [27].

In contrast to CHIKV, other alphaviruses have been demonstrated to activate the NF-κB pathway. Salmonid alphavirus (SAV) has been shown to activate the NF-κB, predominantly via nsP2 expression [28]. Venezuelan equine encephalitis virus (VEEV) has also been shown to activate the NF-κB pathway, and that IKKβ enhances viral replication [29] via phosphorylation of VEEV nsP3, which is required for negative-strand RNA synthesis [30]. In differentiated neuronal cells, Sindbis virus (SINV) infection induced NF-κB activation and, similarly to VEEV, IKKβ was shown to enhance SINV replication [31].

Interestingly, ADP-ribosylation has been implicated in the NF-κB signalling pathway, specifically in the formation of the central IKK complex [32]. ADP-ribosylation of NEMO (NF-κB essential modulator, also referred to as IKK-γ) by ARTD10 inhibits poly-K63 ubiquitination of the protein, blocking the formation of an active IKK complex and concomitantly preventing activation of the NF-κB pathway. More recently, ARTD10 (also referred to as PARP-10) has also been implicated in CHIKV replication, as it can ADP-ribosylate nsP2, inhibiting its proteolytic activity, resulting in reduced viral replication, a process reversable by nsP3-mediated ADP-hydrolysis. We therefore hypothesised that the CHIKV nsP3 macro domain may be able to perturb NF-κB signalling, as a result of its ability to either bind or hydrolyse ADP-ribose. Here, we demonstrate that CHIKV infection does not activate the NF-κB pathway and is unable to suppress exogenous activation of the pathway via TNFα treatment. However, in contrast, we further demonstrate that ectopic expression of nsP3 is able to inhibit both basal and exogenously stimulated activity of the NF-κB pathway, showing that this is likely mediated via the ADP-ribose binding and the hydrolase activities of the N-terminal macro domain.

## 2. Materials and Methods

### 2.1. Cell Culture

A549 cells were maintained at 37 °C with 5% CO_2_ in DMEM supplemented with 10% FCS, 0.5 mM non-essential amino acids, and penicillin-streptomycin (100 units/mL).

### 2.2. Virus Stocks and Infection

Infectious CHIKV was derived from the ECSA genotype LR2006-OPY1 isolate of CHIKV, generated and titred as described previously [33]. Cells were washed with PBS, virus was diluted to the indicated MOI in serum-free DMEM and applied to cells. Cells were rocked for 5 min and incubated for 1 h at 37 °C prior to removal of virus, washed in PBS multiple times, and then the media was replaced with complete media.

### 2.3. Plaque Assays

All plaque assays were performed using BHK-21 cells. Cells were seeded in 12-well plates at a density of 2 × 10^5^ cells/well 16 h prior to assay. Ten-fold serial dilutions of virus-containing supernatant were performed in DMEM. The pre-plated BHK-21 cell media was removed and replaced with 150 µL of relevant virus dilution. Plates were rocked for 5 min at room temperature, then incubated for 1 h at 37 °C. Virus was removed, and the cells washed three times with PBS then the cells were overlaid with 1 mL methyl cellulose (0.8% in complete media). Cells were incubated for 72 h prior to fixation with 10% formaldehyde solution and staining with 0.5% crystal violet. Plates were then washed in water until plaques were visible.

### 2.4. Plasmid Constructs

The expression plasmid for nsP3 (nsP3-F) was generated by PCR amplification of the coding sequence from the LR2006-OPY1 infectious clone plasmid, including flanking restriction sites and a C-terminal FLAG tag, and cloned into pcDNA3.1+ using *Bam*HI and *Not*I restriction sites. The D10A mutant was generated in the nsP3-F plasmid using site-directed mutagenesis (NEB, Q5) (primer sequences available upon request). The GFP plasmid was generated in a similar manner as the nsP3-F construct but lacking the C-terminal FLAG tag. The FLAG-tagged B14 expression plasmid was kindly provided by Geoffrey Smith (University of Cambridge, Cambridge, UK) and has been previously described [34,35]. Plasmids were transfected into cells using Lipofectamine 2000 (Invitrogen (Thermo Fisher Scientific), Waltham, MA, USA).

### 2.5. Luciferase Assays

Cells were transfected with 180 ng of reporter plasmid expressing a firefly luciferase (F-luc) under the control of an NF-κB-sensitive promoter, as previously described [36], alongside 20 ng of transfection control pRL-TK (Promega, Madison, WI, USA), using Lipofectamine 2000 (Life Technologies, Carlsbad, CA, USA). The luciferase samples were collected and quantified using the Dual Luciferase reporter assay system (Promega).

### 2.6. Immunofluorescence

Cells were seeded on coverslips prior to experimentation. Cells were washed with PBS, fixed with 4% paraformaldehyde for 10 min at room temperature, washed with PBS and permeabilised in 0.5% Triton-X-100 for 10 min, washed with PBS, blocked in 2% BSA for 1 h, washed with PBS, and incubated with primary antibodies (rabbit anti-nsP3, 1:5000, kindly provided by Andres Merits, University of Tartu, or mouse anti-p65, 1:50, Santa Cruz SC-8008) for 1 h. After washing in PBS, secondary antibodies (Life Technologies) were applied for 1 h. Coverslips were finally washed with PBS, rinsed in dH_2_O, then mounted in Prolong-gold with DAPI. Cells were imaged using a Zeiss LSM 880 confocal microscope (Jena, Germany) images were processed using Fiji software [37].

### 2.7. Western Blotting

A total of 30 µg of protein in Laemmli buffer was loaded on 10% or 12% SDS-PAGE for B14 and nsP3, respectively. Protein was transferred from gels onto membrane (Immobilon FL, Merck, Darmstadt, Germany) using a semi-dry blotter for 1 h at 15 V. Membranes were blocked using blocking buffer (LICOR) for 20 min at room temperature prior to incubation with primary antibody prepared in TBS (rabbit anti-nsP3, anti-phospho-p105 NEB 4806, anti-Flag Sigma F3165, or anti-actin Sigma A1978), rocking overnight at 4 °C. Membranes were washed in TBS, and secondary antibodies (LICOR) were added for 1 h at room temperature. Membranes were then washed in TBS + 0.1% Tween, then dH_2_O prior to drying and imaging using the LICOR Odyssey scanner (Lincoln, NE, USA).

## 3. Results

### 3.1. CHIKV Infection Does Not Activate the NF-κB Pathway

The NF-κB pathway is activated as part of the innate response to viral infection, leading to the expression of antiviral genes, and is frequently targeted for viral immune evasion [38,39]. Given the importance of this pathway, it is surprising that the interplay between CHIKV infection and the NF-κB pathway is poorly understood, and we sought to address this gap in our knowledge.

**Figure 1:**
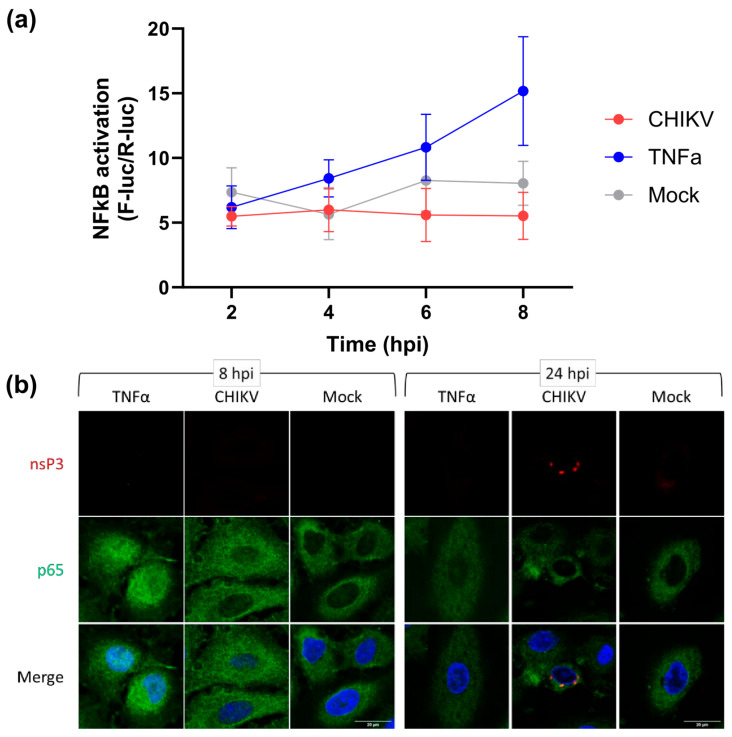
CHIKV infection does not activate the NF-κB pathway. (**a**) A549 cells were transfected with NF-κB reporter plasmids 16 h prior to CHIKV infection (MOI = 5), mock infection, TNFα treatment (50 ng/mL) or mock treatment. Cells were lysed at indicated time points and assayed for luciferase activity. Firefly luciferase values (F-luc) were normalised to Renilla luciferase (R-luc). (n = 9, from three separate experiments). (**b**) Immunofluorescence analysis of NF-κB p65 in infected cells. A549 cells were infected with CHIKV (MOI = 5), mock infected or TNFα treated (50 ng/mL). Cells were fixed at 8 or 24 hpi and stained for p65 (green) and nsP3 (red). Nuclear p65 indicates activation of the NF-κB pathway. Scale bar indicates 20 µm.

We first examined the effect of CHIKV infection on the NF-κB pathway using a reporter plasmid containing firefly luciferase (F-luc) under the control of an NF-κB-sensitive promoter, with a second plasmid, as a transfection control, containing renilla luciferase (R-luc) under the control of the constitutive thymidine kinase (TK) promoter. We chose to use A549 (human lung carcinoma) cells for this analysis, as they can be efficiently infected with CHIKV [40] (Figure S2), are known to be immune-competent [41], and respond well to exogenous (e.g., TNFα) activation of the NF-κB pathway (Figure S3). Following transfection, cells were incubated for 16 h, then either infected with CHIKV (MOI = 5) or treated with TNF-α (50 ng/mL). NF-κB activation was determined by normalising the Fluc values to Rluc. As shown in Figure 1a, CHIKV infected cells showed no increase in NF-κB activation up to 8 h post infection, behaviour which was indistinguishable from that of the negative control (mock-infected in the absence of TNF-α). In contrast, cells treated with TNFα demonstrated a constant increase in NF-κB activation over the time course.

NF-κB activation involves nuclear translocation of the p50-p65 heterodimer (also referred to as p105). A549 cells, either treated with TNFα or infected with CHIKV, were analysed by immunofluorescence using antibodies to p65 to assess NF-κB activation and nsP3 to detect CHIKV infection at either 8 or 24 h post treatment (hpt) or infection (hpi). Figure 1b shows that CHIKV-infected cells exhibited a cytoplasmic localisation of p65 at both 8 and 24 hpi, consistent with the lack of NF-κB activation following CHIKV infection. CHIKV infection was confirmed by the presence of nsP3-positive cells at 24 hpi, however, nsP3 was not detectable at 8 hpi. As expected, TNFα treatment resulted in translocation of a proportion of p65 into the nucleus of cells at 8 hpt, with some protein retained in the cytoplasm. TNFα-mediated activation of NF-κB was transient, as by 24 hpt, p65 was predominantly cytoplasmic, similar to the results for the mock-treated cells. This timescale is consistent with a reduced luciferase signal in TNFα-induced cells by 24 hpt compared to 6 hpt (Figure S3a). These data collectively demonstrate that CHIKV infection does not result in activation of the NF-κB pathway.

**Figure 2:**
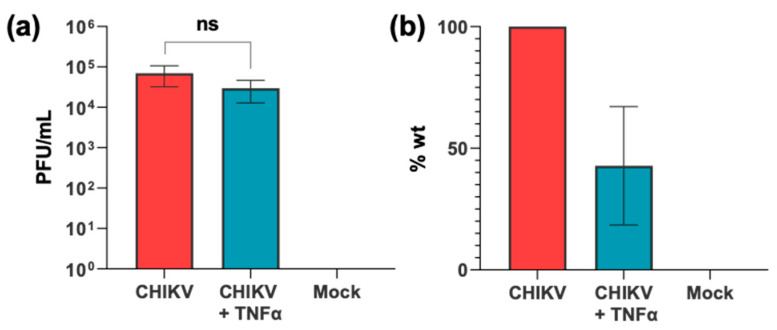
Effect of activating the NF-κB pathway on CHIKV titres. A549 cells were infected with CHIKV (MOI = 5), in the presence or absence of TNFα (50 ng/mL). Supernatant was harvested at 24 h and titred by plaque assay (three separate experiments combined, each at n = 3). Data shown as raw virus titre values (**a**) and as a percentage of wt (**b**).

We proceeded to ask whether exogenous activation of the pathway would have an effect on CHIKV virus production. Treatment of infected A549 cells with TNFα resulted in a modest and non-significant two-fold reduction in virus production, as determined by plaque assay titration of virus released into the culture supernatant (Figure 2). We conclude that exogenous activation of the NF-κB pathway by TNF-α does not significantly inhibit the CHIKV lifecycle and production of progeny virus.

### 3.2. CHIKV Infection Does Not Inhibit Exogenous Activation of the NF-κB Pathway

**Figure 3:**
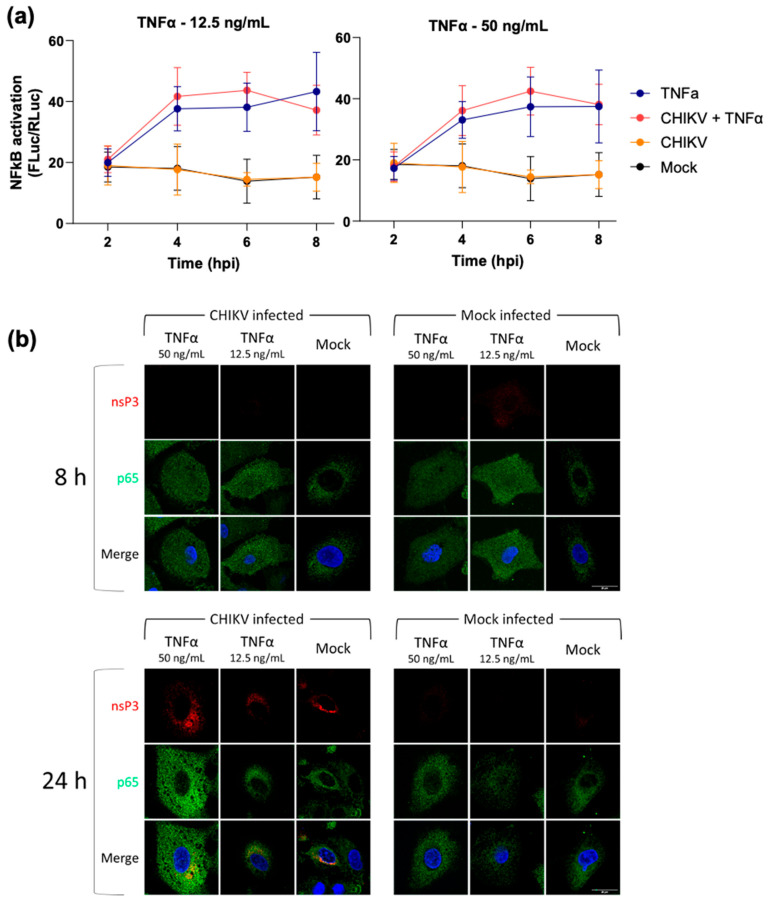
CHIKV infection does not inhibit TNFα-induced NF-κB activation. (**a**) A549 cells were transfected with the NF-κB reporter plasmids, incubated for 16 h prior to either infection with CHIKV (MOI = 5), or mock infected for 1 h, then either TNFα treated (50 ng/mL or 12.5 ng/mL) or mock treated. Cells were lysed at indicated time points and lysates assayed for luciferase. Data shown as F-luc signal normalised to R-luc (n = 3). (**b**) Corresponding A549 cells were fixed at 8 or 24 h p.i. and immunostained for nsP3 (red) and p65 (green), with a DAPI counterstain for nuclei. Scale bar indicates 20 µm.

We proceeded to investigate whether CHIKV infection had any effect on exogenous activation of the NF-κB pathway. To test this, A549 cells were transfected with the NF-κB reporter plasmid and infected with CHIKV at 16 h post-transfection. Immediately after infection, cells were treated with TNFα, and cell lysates were harvested over a 24 h time period. We performed this experiment at two different TNFα concentrations (12.5 and 50 ng/mL) to control for any potential masking of an inhibitory effect at the higher concentration used in previous assays (12.5 ng/mL was the lowest concentration that stimulated luciferase reporter expression, Figure S3b). This analysis revealed that CHIKV infection was not able to inhibit the exogenous activation of NF-κB by TNFα (Figure 3a). To confirm this result, cells were analysed by immunofluorescence for nsP3 and p65. As shown in Figure 3b, CHIKV infection did not block the nuclear translocation of p65 at 8 h following TNFα treatment. Similar to the results shown in Figure 1, by 24 hpt, the stimulatory effect of TNFα treatment had ceased, and the majority of p65 was cytoplasmic. CHIKV infection was confirmed by the presence of nsP3-positive cells at 24 hpi. Taken together, these data demonstrate that CHIKV infection is unable to suppress exogenous activation of the NF-κB pathway by TNFα.

### 3.3. Ectopic Expression of nsP3 in the Absence of Other CHIKV Proteins Inhibits the NF-κB Pathway

**Figure 4:**
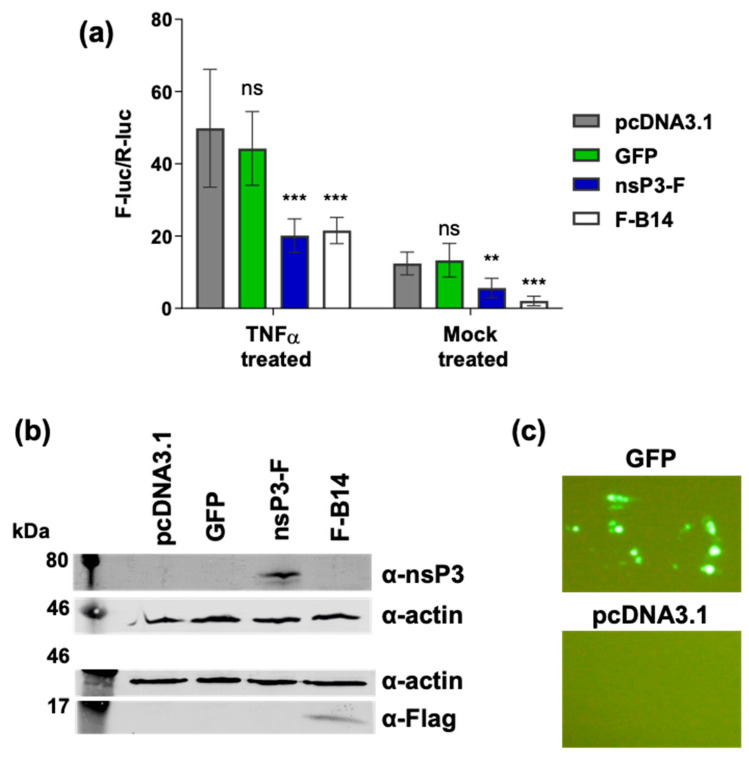
Expression of nsP3 alone inhibits NF-κB activation. (**a**) A549 cells were transfected with the NF-κB reporter plasmids, and co-transfected with expression plasmids for either GFP, FLAG-tagged nsP3 (nsP3-F), FLAG-tagged vaccinia B14 (F-B14) or pcDNA3.1 (empty vector) control. At 16 h p.t. cells were TNFα (50 ng/mL) or mock treated for 6 h prior to lysis, and assayed for luciferase activity (two separate experiments combined, each n = 3, data analysed by One-way ANOVA with Bonferroni correction compared to empty vector). ns (*p* > 0.05), ** (*p* < 0.01) or *** (*p* < 0.001). (**b**) Western blot for nsP3-F (59 kDa) and F-B14 (15 kDa). (**c**) Wide field fluorescent microscopy images of GFP-expressing cells compared to cells transfected with empty vector.

We originally hypothesised that by virtue of its ADP-ribosyl-binding and hydrolase activities, the CHIKV nsP3 macro domain might be able to modulate NF-κB activation. The observation that CHIKV infection did not activate NF-κB suggested that the virus possessed a mechanism to block endogenous activation of the pathway following infection. However, it was conceivable that any subtle and perhaps transient effect of the macro domain might be obscured by the rapid replication and subsequent cytotoxicity of virus infection. To formally determine whether the macro domain could function to modulate NF-κB activation, we assessed whether any effect could be observed when nsP3 was expressed in the absence of other viral proteins. To do this, a C-terminally FLAG-tagged nsP3 expression construct was generated (nsP3-F). As a positive control, we utilised N-terminally FLAG-tagged vaccinia B14 (F-B14). B14 is a potent and well characterised inhibitor of the NF-κB pathway, it binds IKKβ and blocks formation of an active IKK complex [34]. Cells were transfected with NF-κB reporter plasmids, together with either expression constructs for nsP3-F, F-B14 or GFP, with or empty vector plasmid, and incubated for 16 h, prior to treatment with TNFα for an additional 6 h. As shown in Figure 4a, both nsP3 and B14 exhibited a similar inhibitory effect on NF-κB activity, both basal levels and the levels of NF-κB exogenously activated via TNFα. The expression of GFP exerted no significant effect on NF-κB activation. The expression of nsP3, B14 was confirmed by Western blotting (Figure 4b), and GFP expression was confirmed by IF (Figure 4c).

**Figure 5:**
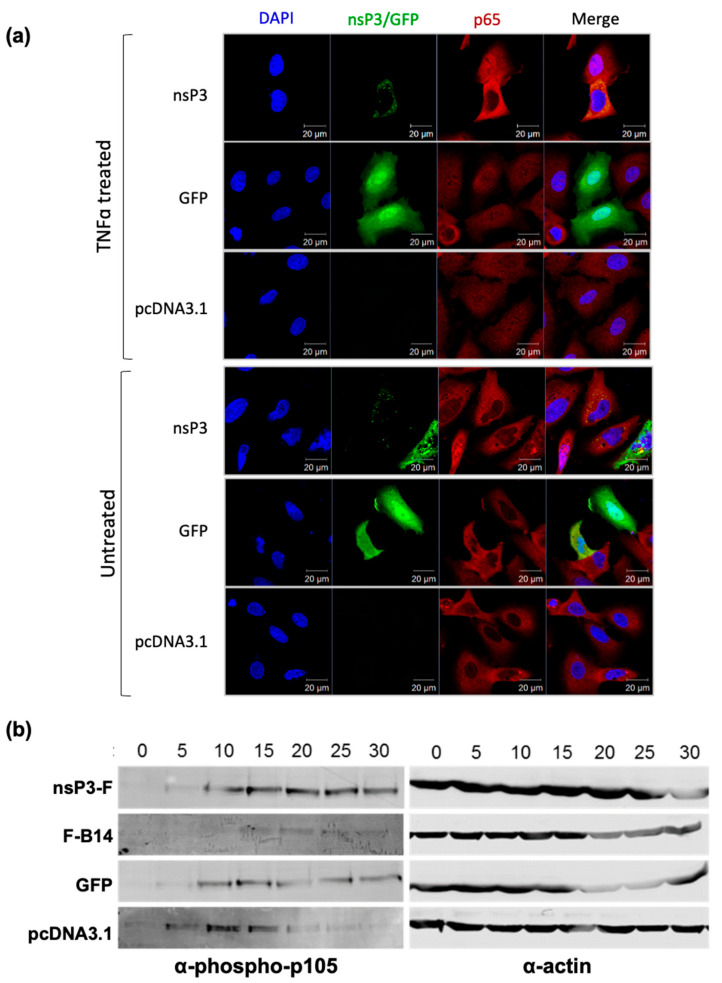
Expression of nsP3 alone blocks nuclear translocation of NF-κB but does not inhibit IKK complex activation (**a**) A549 cells were transfected with expression plasmids for either nsP3-F, GFP or pcDNA3.1 (empty vector control). At 16 h p.t., cells were treated with TNFα (50 ng/mL) or mock treated for 6 h prior to fixation and staining for p65 (red). Cells were co-stained for nsP3 (shown in green), except GFP expressing cells. (**b**) A549 cells were transfected with expression plasmids for nsP3-F, F-B14, GFP, or pcDNA3.1 (empty vector control). Cells were incubated for 16 h then activated with TNFα (50 ng/mL). Cells were lysed at 5 min intervals over a 30 min period, and western blotting was performed for the detection of phospho-p105 and actin as a loading control. The presence of phospho-p105 indicates an active IKK complex.

To confirm these findings, we again performed immunofluorescence to determine the subcellular localisation of the p65 subunit of NF-κB. As demonstrated in Figure 5a, TNFα treatment resulted in nuclear translocation of p65 in cells transfected with empty vector or GFP. Conversely, p65 was excluded from the nucleus in cells expressing nsP3-F, indicating that nsP3 was inhibiting the nuclear translocation of NF-κB and therefore, the activation of the pathway.

To further determine at which stage in the NF-κB pathway nsP3 may be acting, we performed a Western blot analysis for phosphorylated-p105, which is mediated rapidly by the IKK complex after the pathway is stimulated (Figure S4). As shown in Figure 5b, in the mock (pcDNA3.1 empty-vector transfected) cells, phospho-p105 is detectable as early as 5 min post treatment, persisting for approximately 15 min and declining thereafter. In the GFP-expressing cells, there is a slight delay in phospho-p105 detection, compared to that for the empty-vector control, by about 5 min. This is similar to the results for the nsP3-F expressing cells. In cells expressing vaccinia B14, a protein known to inhibit the IKK complex, phospho-p105 is weakly detectable at 15–30 min post-treatment. This is as expected, as B14 directly inhibits the IKK complex via an interaction with IKKβ. Collectively, these data imply that nsP3-F is not blocking IKK activation but inhibits the nuclear translocation of p65/p105 to the nucleus.

### 3.4. The nsP3 Macro Domain Contributes to Inhibition of NF-κB Activation

**Figure 6:**
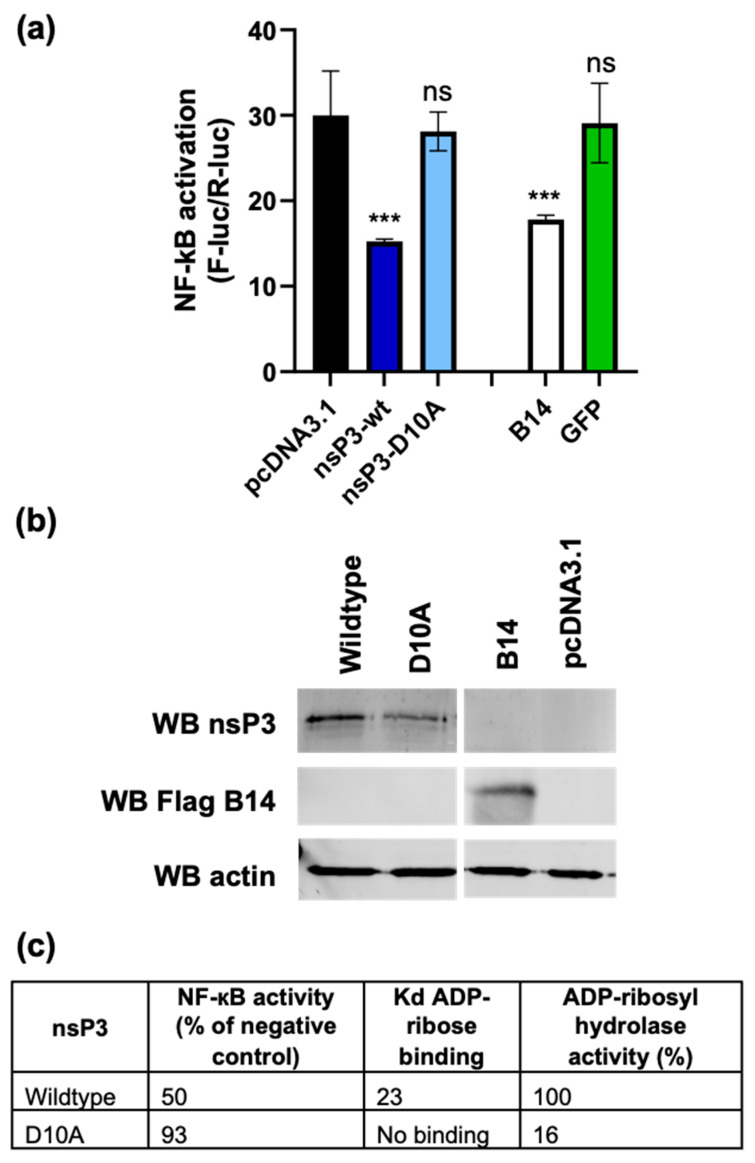
The nsP3 macro domain contributes to inhibition of NF-κB activation. (**a**) A549 cells were transfected with the NF-κB reporter plasmids and either pcDNA3.1 (empty vector control), or WT- or D10A- nsP3-F, F-B14 or GFP. At 16 h p.t., cells were TNFα activated (50 ng/mL) prior to lysis at 6 h post-treatment and assayed for luciferase (n = 3, data analysed by One-way ANOVA with Bonferroni correction compared to empty vector control). ns (*p* > 0.05), or *** (*p* < 0.001). (**b**) Confirmation of protein expression by western blotting for nsP3 and Flag tag (for F-B14) on the corresponding cell lysates. (**c**) Values for ADP-ribose binding and ADP-ribosyl hydrolase activity taken from [20] compared to NF-κB inhibition from Figure 6a (% of negative control).

To assess whether the macro domain was involved with the nsP3-mediated inhibition of the NF-κB pathway, we compared the nsP3 mutant D10A which has been previously shown to be critical for ADP-ribose binding and hydrolysis [20] (Figure 6c). Mutant and wt sP3-F expression constructs, control F-B14 and GFP constructs, or empty vector plasmid were transfected with NF-κB reporter plasmids into A549 cells, as described above. As shown by Figure 6a, the nsP3-D10A mutant had no significant effect on NF-κB activation, indistinguishable from the results for the negative controls, implying that the inhibition of the NF-κB pathway is mediated by the macro domain. The expression levels for wt and mutant nsP3, as well as for the B14 control, were confirmed by Western blotting (Figure 6b).

**Figure 7:**
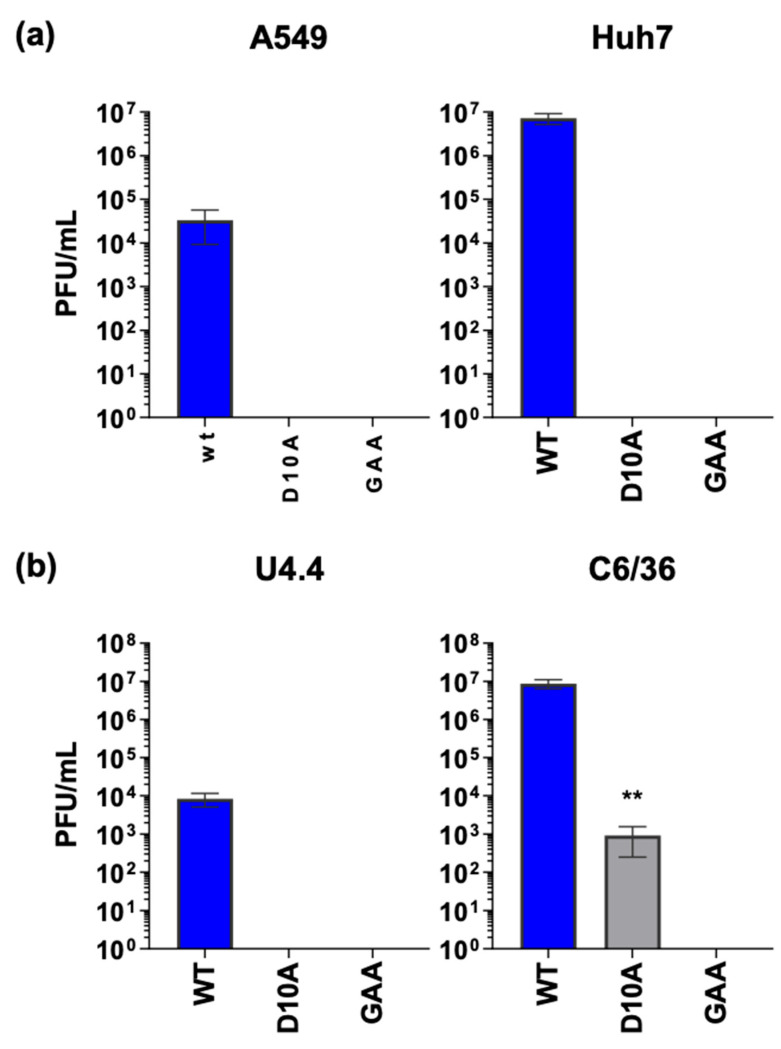
Phenotype of nsP3 macro domain mutant D10A in the context of infectious virus in mammalian and insect cell lines. Indicated cell lines were either electroporated (mammalian cells (**a**)) or transfected by lipofection (insect cells (**b**)) with either WT, D10A, or GAA (RdRp-defective) ICRES CHIKV RNA. Supernatant was collected from cells at either 24 h p e (for mammalian cells) or 48 h p t (for insect cells) and virus titre quantified by plaque assay on BHK-21 cells. ** (*p* < 0.01).

To attempt to correlate the ability of the macro domain to inhibit NF-κB activation with its role in the virus lifecycle, we evaluated the phenotype of the D10A mutant in the context of infectious CHIKV. We tested the replication of the mutant in four cell types: A549, Huh7 (human hepatoma cells), U4.4, and C6/36 (both *Aedes albopictus*). As a negative control, we included an inactivating mutant in the nsP4 RNA-dependent RNA polymerase (GAA) for each cell type. In both mammalian cell lines (A549 and Huh7), the D10A mutant was unable to produce virus (Figure 7a). Similarly, the D10A mutant was unable to replicate in the *Aedes albopictus* cell line U4.4 (Figure 7b). Intriguingly, in the C6/36 cells (Figure 7b), which lack a functional RNAi response due to a frameshift mutation in the Dcr2 gene [42], D10A, which was completely defective in all other cell lines, was able to produce infectious virus, suggestive of an additional role of the macro-domain in counteracting the insect antiviral response.

Taken collectively, the mutant nsP3 data suggest that the macro domain not only plays a role in counteracting the mammalian NF-κB pathway but also has a potential role in subverting the insect anti-viral response. However, we should note that the dramatic phenotype of the D10A mutant in both mammalian and insect cells is consistent with the additional roles of the nsp3 macrodomain in virus replication.

## 4. Discussion

The transcription factor NF-κB is involved in the host cell defence against virus infection, and its activity is induced via a variety of canonical and non-canonical pathways [38], in particular, pattern recognition receptors (PRRs) involved in the innate immune detection of virus components. Virus infection is a well-characterised inducer of NF-κB activity, and most viruses have evolved mechanisms of inhibiting this activation to evade this response, a good example is vaccinia virus, which expresses no less than nine different proteins that antagonise the NF-κB pathway [43].

In this study, we sought to investigate the effect of CHIKV infection on NF-κB activity. We observed that CHIKV infection could neither induce the pathway nor inhibit activation via the canonical pathway as a response to the external stimulus provided by TNFα treatment. We reasoned that the lack of NF-κB activation during infection was consistent with the hypothesis that CHIKV was able to inhibit NF-κB activation, but that this block could be overcome by exogenous stimulation (e.g., by TNFα–Figure 3). A likely mediator of this effect was the macro domain of nsP3, and indeed, the ectopic expression of nsP3 alongside mutant D10A, which had previously been characterised to be unable to bind or hydrolyse ADP-ribose [20], revealed that this was indeed the case. The lack of NF-κB inhibition demonstrated by the D10A mutant suggests that the ADP-ribose binding and hydrolysis properties of nsP3 are responsible for this effect, however, further research is required to precisely determine the mechanism of inhibition. It is also formally possible that other domains of nsP3 play a role in the regulation of the NF-κB pathway, and further mutagenesis should be undertaken to investigate this possibility.

At this stage in our investigation, we do not know at which point nsP3 is antagonising the NF-κB pathway. The phosphorylation of p105 is mediated by an active IKK complex, thus, one attractive hypothesis is that the nsP3 macro domain is perturbing the ADP-ribosylation of NEMO, a key component of the IKK complex. Consistent with this, a cellular mono-ADP-ribosyltransferase, ARTD10 (PARP10), inhibits TNFα mediated NF-κB activation by interfering with the poly-K63 ubiquitinylation of NEMO [32,44]. Indeed, both NEMO and ARTD10 were shown to be in vitro substrates for the ADP-ribosylhydrolase activity of the nsP3 macro domain [44] and were efficiently de-mono-ADP-ribosylated (deMARylated). In this study, however, we show that ectopically expressed nsP3 inhibited the NF-κB pathway but not at the stage of IKK complex formation (Figure 5b). A recent study showed that the nsP3 macro domain antagonises the ADP-ribosylation of nsP2 by ARTD10 [21], indicating that there may be multiple targets of ADP-ribosylation-mediated regulation in innate anti-viral pathways for nsP3 to counteract. Although we have been unable to biochemically demonstrate an interaction between nsP3 and either NEMO or ARTD10, we did observe partial co-localisation of nsP3 with ARTD10 during the late stages of CHIKV infection (Figure S5). This is consistent with the hypothesis that nsP3 is able to interfere with ARTD10 function in infected cells. Therefore, we propose that the nsP3 macro domain directly interacts with one or more ADP-ribosylated proteins involved in the NF-κB pathway. However, given the previous observation that miR-146a is also involved in the regulation of the NF-κB pathway by CHIKV, we believe it is highly likely that CHIKV, like many other viruses, possesses multiple mechanisms to control this key signalling pathway.

Interestingly, components of the NF-κB pathway have been shown to be pro-viral factors for other alphaviruses. Contrary to our findings for CHIKV, SAV, VEEV, and SINV have all been shown to activate the NF-κB pathway [28,30,31]. Although in this study, we show CHIKV nsP3 to be an inhibitor of the NF-kB pathway, it would be prudent to investigate whether any NF-κB-pathway proteins are pro-viral for CHIKV, particularly as we demonstrated that the pathway is inhibited at a stage following IKK-complex activation.

## 5. Conclusions

To conclude, we show here that one of the roles of the CHIKV nsP3 macro domain is to contribute to the regulation of a critical antiviral factor, the transcription factor NF-κB. This observation leads to a number of key questions for future research, e.g., is this function conserved in other viral macro domains? What are the other targets for the ADP-ribosylhydrolase activity of these domains? And indeed, why is the macro domain only present in a small subset of positive-strand RNA viruses? What is clear is that we have only just begun to understand the role of this domain in the lifecycle of these viruses.

## Data Availability

The original contributions presented in this study are included in the article/Supplementary Material. Further inquiries can be directed to the corresponding author.

## Supplementary Materials

The following supporting information can be downloaded at: www.mdpi.com/article/10.3390/v17020191/s1, Figure S1: nsP3 and the macro domain; Figure S2: CHIKV virus production in a range of cell lines; Figure S3: Optimisation of TNFα treatment in A549 cells; Figure S4: Schematic of IKK complex activation; Figure S5: ARTD10 colocalization with nsP3. Refs. [45,46,47] are cited in the supplementary materials.
