# Supplementary material for: The Chikungunya Virus nsP3 Macro Domain Inhibits Activation of the NF-κB Pathway
Source: Viruses. 2025 Jan 29;17(2):191. doi: 10.3390/v17020191 (PMC11861268; doi:10.3390/v17020191)
Supplement: Supplementary file 1 [file viruses-17-00191-s001.zip › viruses-3354263-supplementary.pptx]

## Slide 1
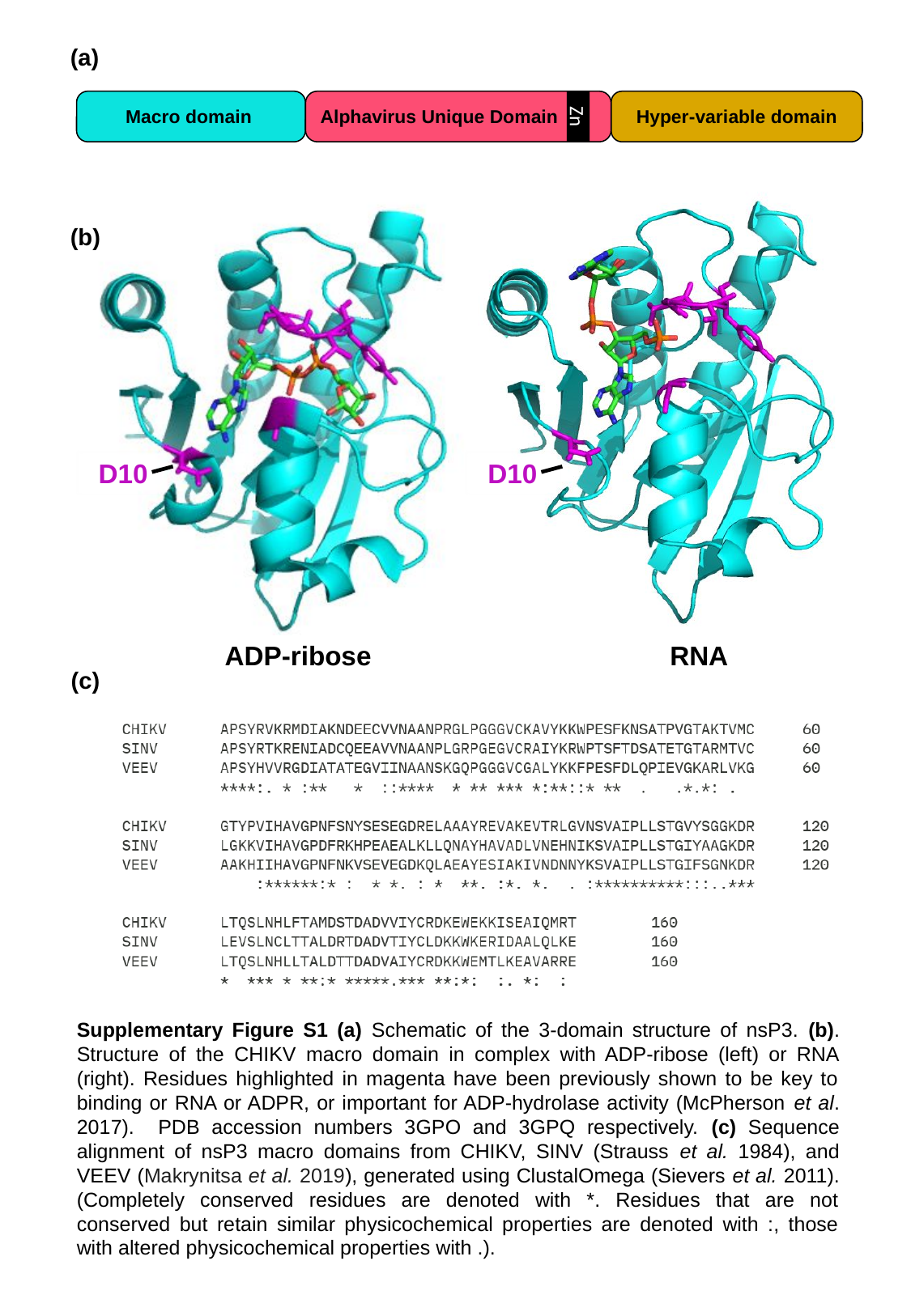

(a)
Zn
Macro domain
Alphavirus Unique Domain
Hyper-variable domain
(b)
D10
D10
ADP-ribose
RNA
(c)
Supplementary Figure S1 (a) Schematic of the 3-domain structure of nsP3. (b). Structure of the CHIKV macro domain in complex with ADP-ribose (left) or RNA (right). Residues highlighted in magenta have been previously shown to be key to binding or RNA or ADPR, or important for ADP-hydrolase activity (McPherson et al. 2017). PDB accession numbers 3GPO and 3GPQ respectively. (c) Sequence alignment of nsP3 macro domains from CHIKV, SINV (Strauss et al. 1984), and VEEV (Makrynitsa et al. 2019), generated using ClustalOmega (Sievers et al. 2011). (Completely conserved residues are denoted with *. Residues that are not conserved but retain similar physicochemical properties are denoted with :, those with altered physicochemical properties with .).

## Slide 2
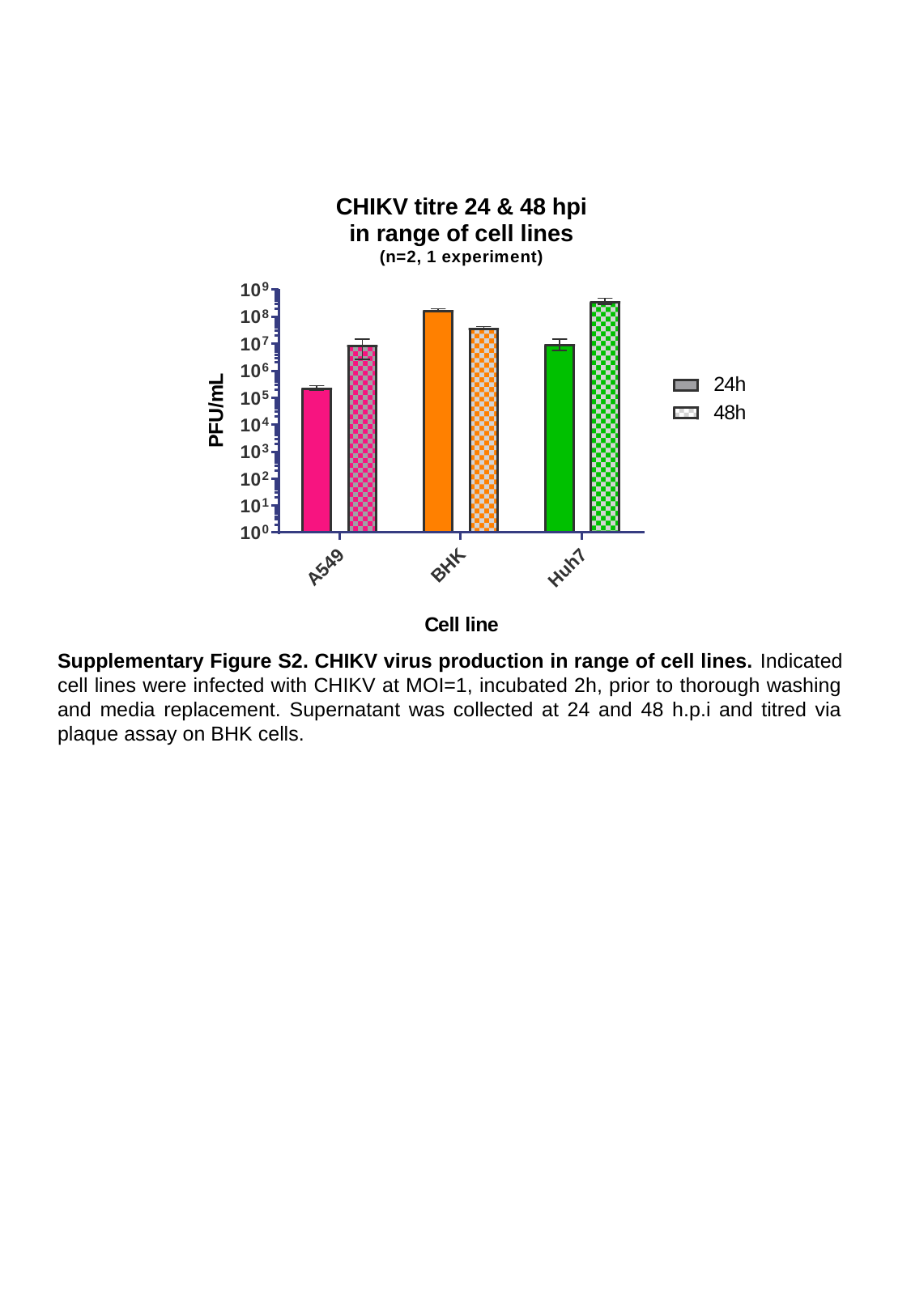

Supplementary Figure S2. CHIKV virus production in range of cell lines. Indicated cell lines were infected with CHIKV at MOI=1, incubated 2h, prior to thorough washing and media replacement. Supernatant was collected at 24 and 48 h.p.i and titred via plaque assay on BHK cells.

## Slide 3
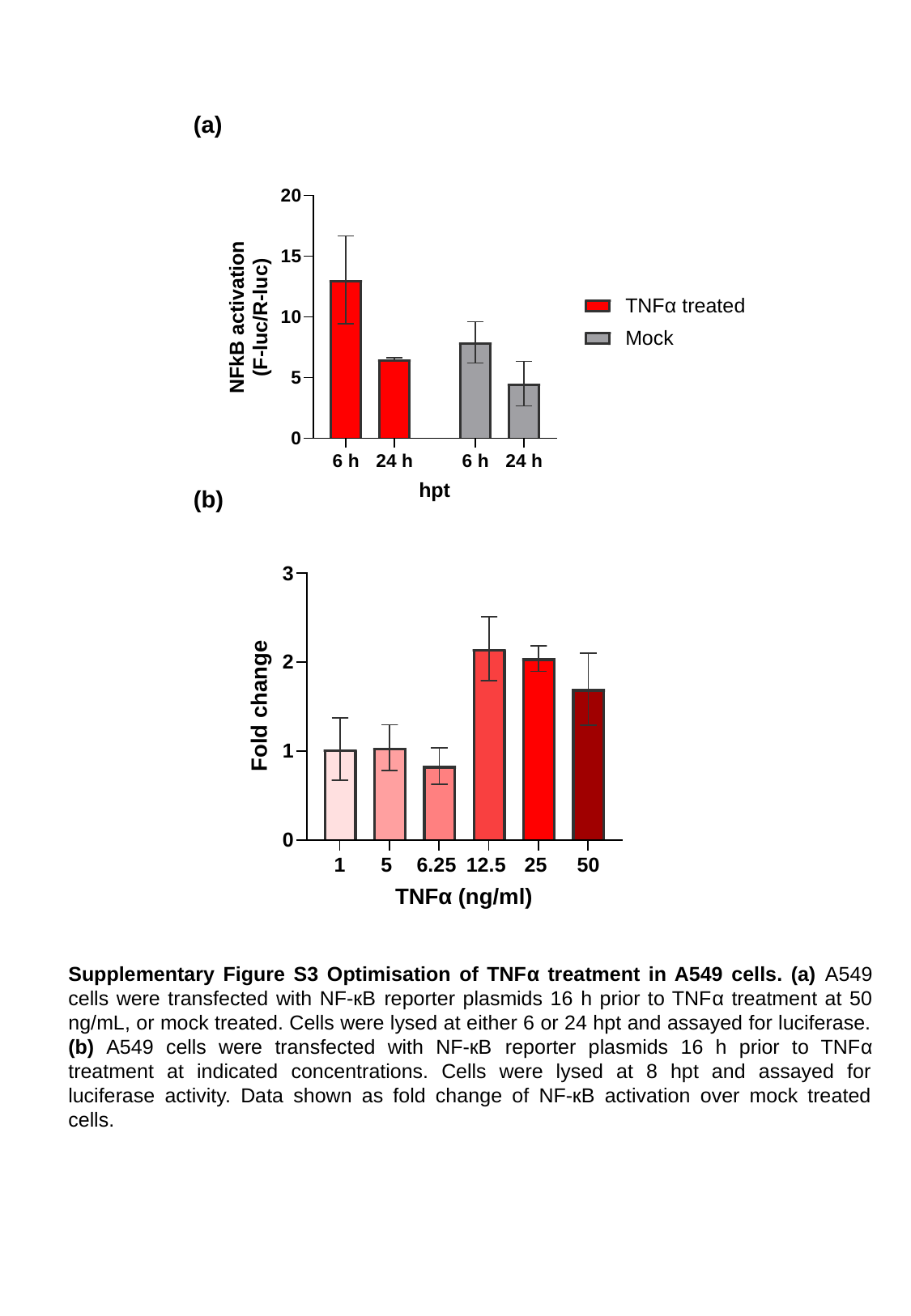

(a)
(b)
Supplementary Figure S3 Optimisation of TNFα treatment in A549 cells. (a) A549 cells were transfected with NF-кB reporter plasmids 16 h prior to TNFα treatment at 50 ng/mL, or mock treated. Cells were lysed at either 6 or 24 hpt and assayed for luciferase. (b) A549 cells were transfected with NF-кB reporter plasmids 16 h prior to TNFα treatment at indicated concentrations. Cells were lysed at 8 hpt and assayed for luciferase activity. Data shown as fold change of NF-кB activation over mock treated cells.

## Slide 4
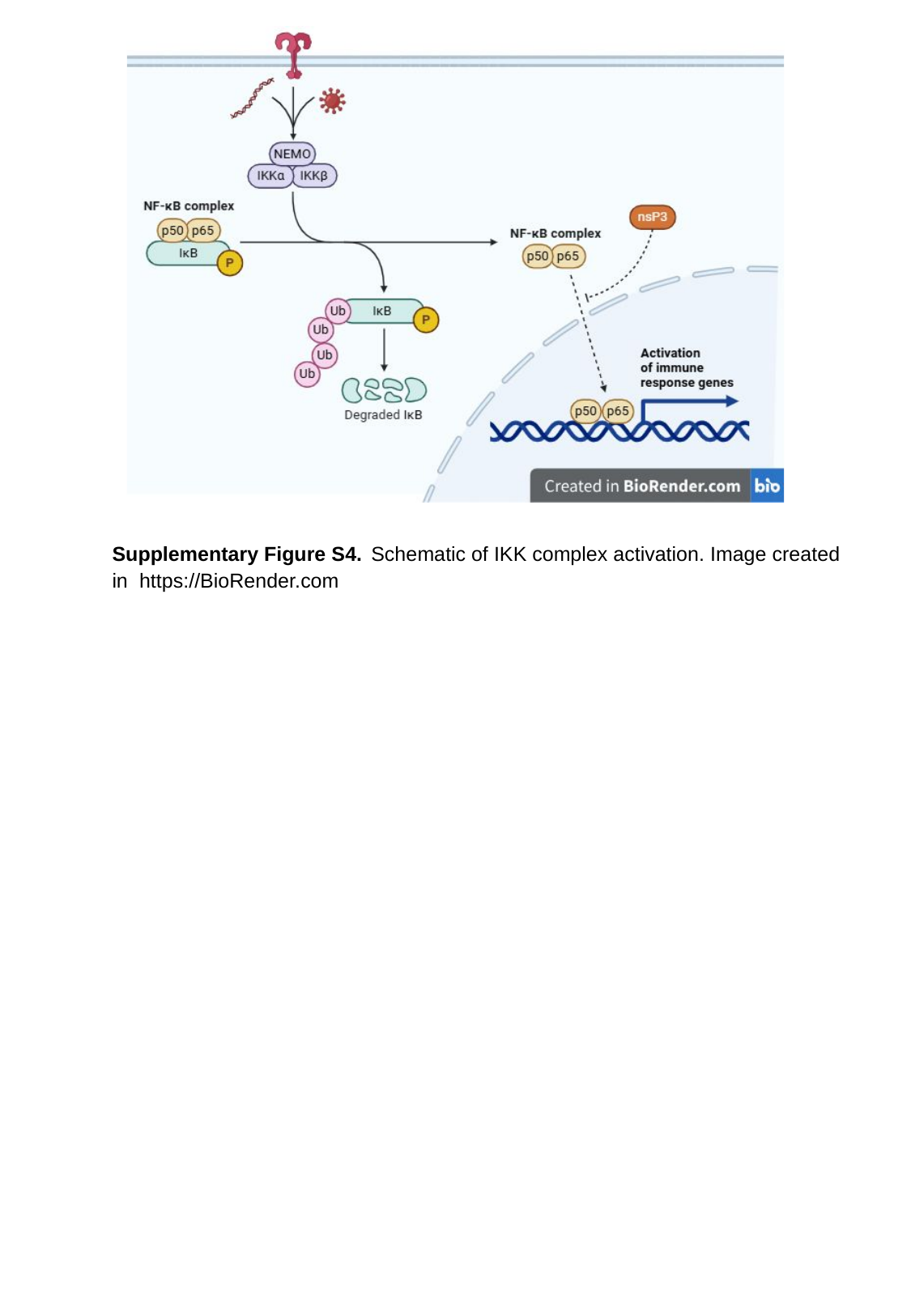

Supplementary Figure S4. Schematic of IKK complex activation. Image created in https://BioRender.com

## Slide 5
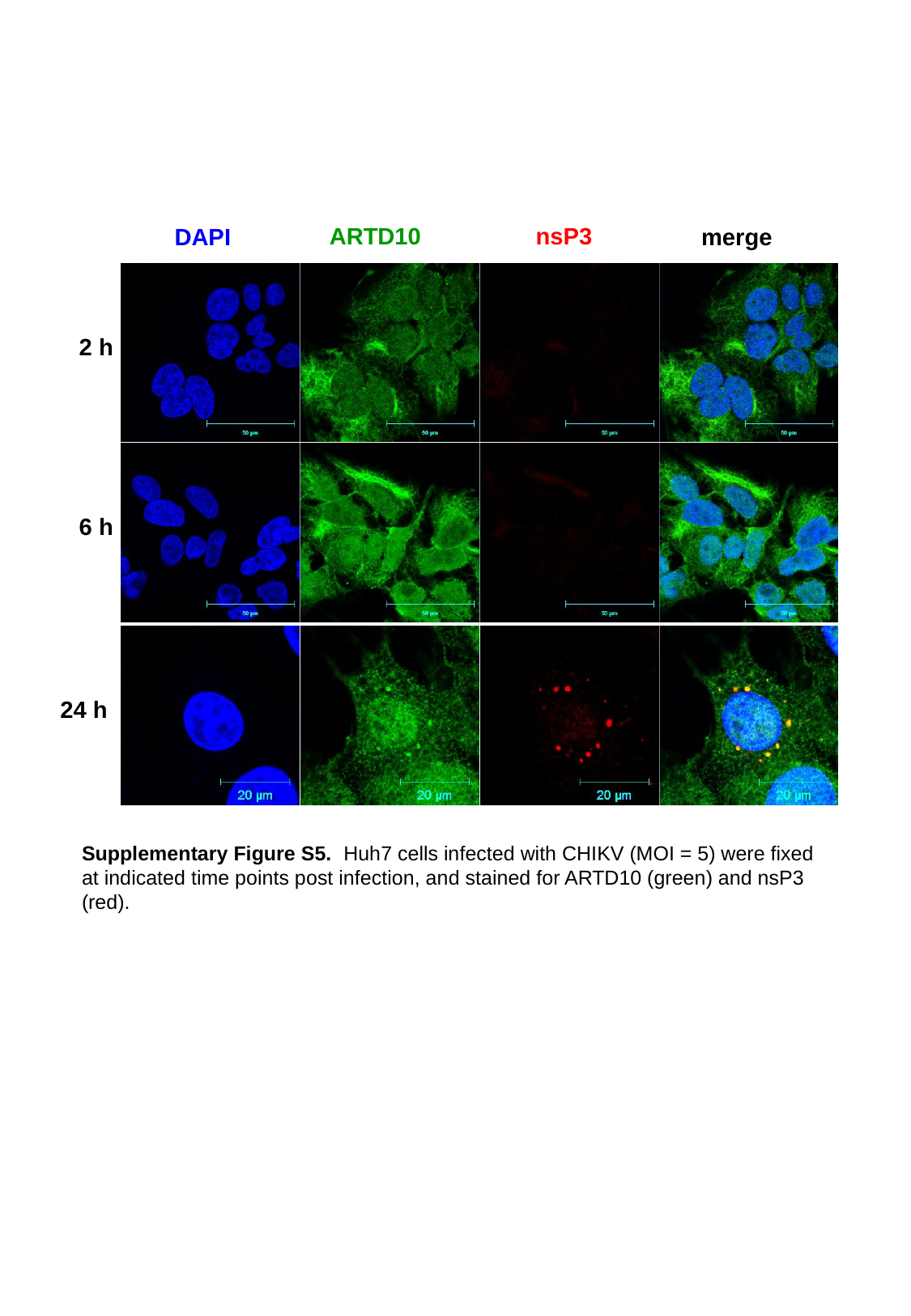

ARTD10
nsP3
merge
DAPI
2 h
6 h
24 h
Supplementary Figure S5. Huh7 cells infected with CHIKV (MOI = 5) were fixed at indicated time points post infection, and stained for ARTD10 (green) and nsP3 (red).

## Slide 6
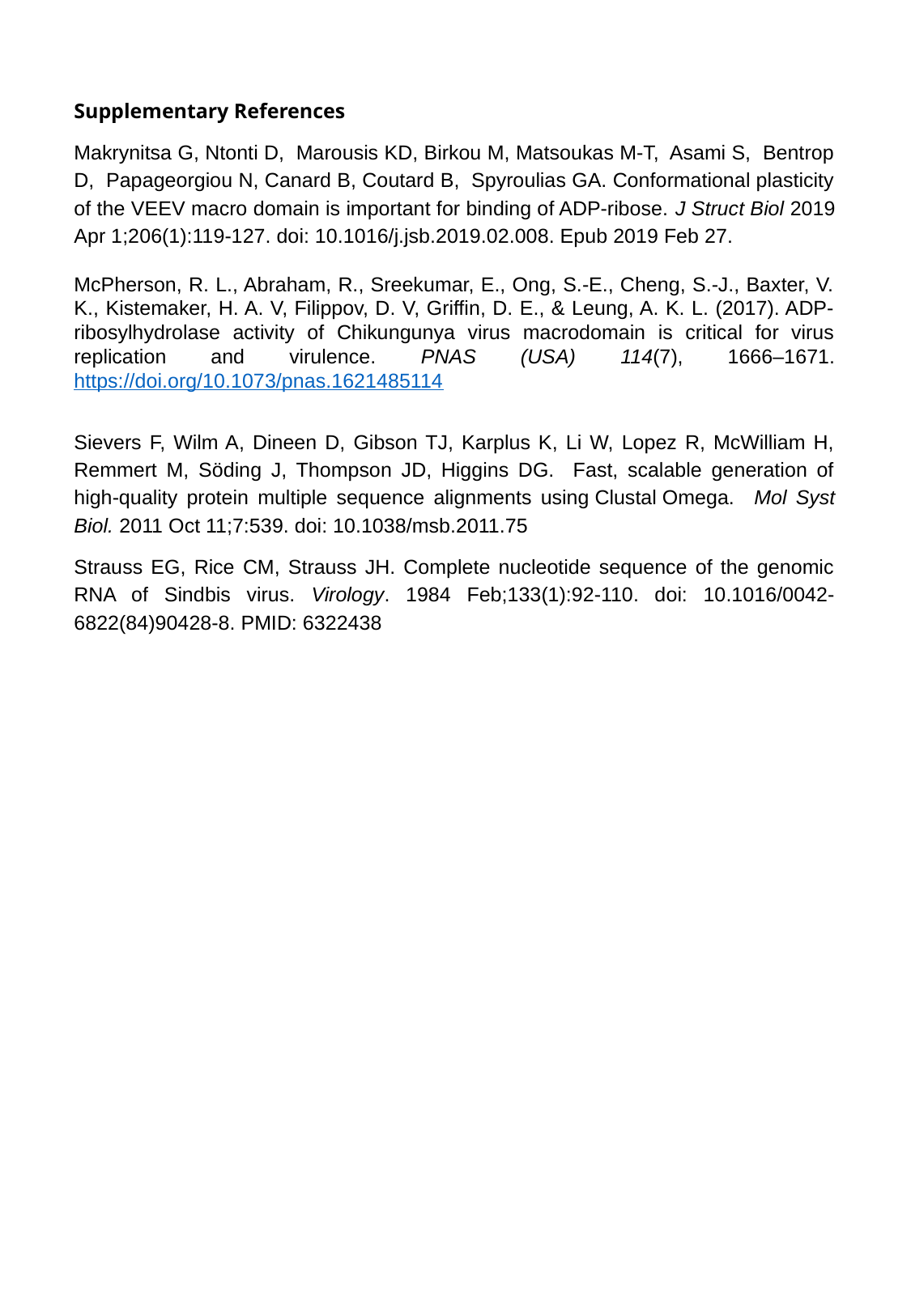

Supplementary References
Makrynitsa G, Ntonti D, Marousis KD, Birkou M, Matsoukas M-T, Asami S, Bentrop D, Papageorgiou N, Canard B, Coutard B, Spyroulias GA. Conformational plasticity of the VEEV macro domain is important for binding of ADP-ribose. J Struct Biol 2019 Apr 1;206(1):119-127. doi: 10.1016/j.jsb.2019.02.008. Epub 2019 Feb 27.
McPherson, R. L., Abraham, R., Sreekumar, E., Ong, S.-E., Cheng, S.-J., Baxter, V. K., Kistemaker, H. A. V, Filippov, D. V, Griffin, D. E., & Leung, A. K. L. (2017). ADP-ribosylhydrolase activity of Chikungunya virus macrodomain is critical for virus replication and virulence. PNAS (USA) 114(7), 1666–1671. https://doi.org/10.1073/pnas.1621485114
Sievers F, Wilm A, Dineen D, Gibson TJ, Karplus K, Li W, Lopez R, McWilliam H, Remmert M, Söding J, Thompson JD, Higgins DG. Fast, scalable generation of high-quality protein multiple sequence alignments using Clustal Omega. Mol Syst Biol. 2011 Oct 11;7:539. doi: 10.1038/msb.2011.75
Strauss EG, Rice CM, Strauss JH. Complete nucleotide sequence of the genomic RNA of Sindbis virus. Virology. 1984 Feb;133(1):92-110. doi: 10.1016/0042-6822(84)90428-8. PMID: 6322438
